# Entrepreneurship in stoma care nursing: difficulties experienced and strategies for overcoming them

**DOI:** 10.1590/0034-7167-2024-0242

**Published:** 2026-08-07

**Authors:** Lívia Nunes Rodrigues Leme, Carolina Cabral Pereira da Costa, Eloá Carneiro Carvalho, Samira Silva Santos Soares, Bruno de Sousa Pappalardo, Gustavo Assis Afonso, Lana de Medeiros Escobar, Norma Valéria Dantas de Oliveira Souza

**Affiliations:** I Universidade do Estado do Rio de Janeiro. Rio de Janeiro, Rio de Janeiro, Brazil; II Universidade Estadual de Santa Cruz. Ilhéus, Bahia, Brazil

**Keywords:** Nursing, Stomatherapy, Entrepreneurship, Professional Autonomy, Job Market., Enfermería, Estomaterapia, Emprendimiento, Autonomía Profesional, Mercado de Trabajo.

## Abstract

**Objectives:**

to analyze the difficulties experienced by stoma therapy nurses in carrying out entrepreneurial activities in the specialty and to discuss strategies for overcoming them.

**Methods:**

a qualitative, descriptive-exploratory study, conducted with 26 entrepreneurial stoma therapists, recruited through the snowball technique. The information was analyzed using content analysis.

**Results:**

difficulties included low professional appreciation and recognition, lack of awareness of the specialty among other healthcare professionals, and limited familiarity with the legal and administrative processes for starting a business. Financial issues and the content gap in training also proved to be significant challenges. The strategies included exchange of experiences among professionals, expansion of the content offered on the topic, and broadening professionals’ perspective on entrepreneurship possibilities.

**Final Considerations:**

it is urgent to implement strategies to overcome the barriers to entrepreneurship in stoma therapy. Such a measure can increase the possibility of professional success, visibility, and appreciation of nursing.

## RESUMEN

**Objetivos:**

analizar las dificultades que experimentan enfermeras de terapia enterostomal al emprender actividades empresariales en la especialidad y discutir estrategias para superarlas.

**Métodos:**

estudio cualitativo, descriptivo-exploratorio, realizado con 26 enfermeras emprendedoras de terapia enterostomal, reclutadas mediante muestreo en cadena. Los datos se analizaron mediante análisis de contenido.

**Resultados:**

las dificultades fueron el escaso reconocimiento y aprecio profesional, el desconocimiento de la especialidad entre otros profesionales sanitarios y la limitada familiaridad con los procesos legales y administrativos para el ejercicio de esta práctica. Los problemas financieros y deficiencias en el contenido de la formación también resultaron ser desafíos importantes. Las estrategias fueron intercambio de experiencias entre profesionales, ampliación del contenido ofrecido sobre el tema y ampliación de la visión de los profesionales sobre las posibilidades empresariales.

**Consideraciones Finales:**

es urgente implementar estrategias para superar las barreras al emprendimiento en el cuidado de ostomías. Esta medida puede aumentar las posibilidades de éxito profesional, visibilidad y reconocimiento de la enfermería.

## INTRODUCTION

Historically, nursing has faced challenges in terms of social recognition, reflecting a hospital-centric and physician-centered model^(1,2)^. However, since 2003, there has been an average annual growth of 25% in the number of establishments managed by nurses, especially private practices, specialized clinics, and home care services in the South and Southeast regions^(3)^.

This advancement in the profession indicates a transition towards interdisciplinarity and a reconfiguration of professional roles, reinforcing the need for more horizontal and collaborative practices for the benefit of individuals, families, and communities^(4)^. Despite this, creative and entrepreneurial nurses still face cultural resistance, often being perceived as merely procedural, which limits their social and professional recognition^(5)^.

It is noteworthy that acting in an innovative and entrepreneurial manner in nursing can be an opportunity to reshape the profession due to its potential to increase professional autonomy and visibility of nursing. Furthermore, it is characterized as a way to meet the population’s health needs in a differentiated, relevant, and timely manner^(6-8)^.

Professional autonomy refers to nurses’ ability to make decisions and conduct their practice based on technical, scientific, ethical, and legal knowledge, encompassing care, management, and health education. Entrepreneurial nurses apply their clinical and managerial knowledge by developing products, technologies, and strategies to improve processes and care^(9)^.

Globally, entrepreneurship in nursing is growing by combining innovation, autonomy, and social impact. Professionals have been exploring niches such as specialized clinics, home care, telenursing, startups, consulting, and digital solutions^(10,11)^. Such initiatives, based on evidence and ethics, strengthen the sustainability of healthcare systems, reposition nursing as a key player, and promote improvements in care and patient experience^(11)^.

Entrepreneurship can be understood as a process in which an individual sees an opportunity and acts upon it to create value, developing something new or improving something that already exists^(8,12)^. The literature classifies entrepreneurship into three types: business entrepreneurship, which refers to the creation of a company or business for autonomous professional practice^(13,14)^; intrapreneurship, in which entrepreneurship is developed within public or private organizations where the entrepreneur already works^(7,8,14)^; and the social aspect, which focuses on using market-based strategies to generate social benefit^(7,8,14)^.

Considering these definitions and the characteristics of the entrepreneurial profile, it is observed that nurses, whether generalists or specialists, can act entrepreneurially in all three modalities. In this context, stoma therapy stands out as a promising specialty, focused on the care of people with stomas, wounds, anal and urinary incontinence, fistulas, tubes and drains^(15,16)^, which opens up wide possibilities for innovative practices.

Stoma therapy has been growing in Brazil, predominantly in the area of wound care, followed by ostomies and incontinence, with a higher concentration of professionals in the Southeast, Northeast, and South regions, where there are more training courses available^(17)^. This scenario fosters entrepreneurship in areas such as doctor’s offices, clinics, home care, consulting, training, and the development of assistive technologies^(17-19)^.

In Brazil, research conducted in 2016 estimated that there were 1,371 specialists trained in courses accredited by the Brazilian Association of Stoma therapy: Stomas, Wounds and Incontinence (In Portuguese, *Associação Brasileira de Estomaterapia: Estomias, Feridas e Incontinências*)^(17)^. Internationally, stoma therapy is also establishing itself as a strategic specialty in healthcare, although there is still a scarcity of data on the workforce in different countries. In the United Kingdom, it is estimated that around 600 specialist nurses work to care for more than 176,000 people with stomas, and in Spain, almost half of public hospitals have stoma therapists^(20,21)^.

Stoma therapists have a wide range of entrepreneurial opportunities, encompassing areas such as assistance, teaching, research, management, advisory services, and technical consulting, in both public and private services, including home care, outpatient clinics, and medical-hospital product companies^(15,16)^. However, although the field is promising, significant barriers still exist, such as structural, bureaucratic, and cultural limitations, which can hinder or discourage the adoption of entrepreneurial practices by these professionals^(5,12)^.

From this perspective, it is necessary to study the situations that hinder the practice of entrepreneurship in nursing, as well as to delve into strategies for overcoming obstacles to the development of entrepreneurial actions in the profession, especially in the specialty in question.

It is noteworthy that discussions about entrepreneurship in nursing are still underexplored, highlighting gaps in the approach to this topic and, even more so, in ostomy care^(8,22,23)^. Thus, the study is relevant because it encourages reflection and debate, strengthening the entrepreneurial and innovative culture in nursing and ostomy care.

This article is a product of one of the authors’ dissertations entitled “*Empreendedorismo na enfermagem em estomaterapia: potencializando oportunidades de trabalho*”^(24)^.

## OBJECTIVES

To analyze the difficulties experienced by stoma therapy nurses in carrying out entrepreneurial activities in the specialty and to discuss strategies for overcoming them.

## METHODS

### Ethical aspects

In order to comply with Resolution 466 of December 12, 2012 of the Brazilian National Health Council, this study was approved by the Research Ethics Committee. Before each interview, participants read, signed, and received a copy of an Informed Consent Form. In order to protect the confidentiality of interviewees’ identities, a coding system was created using the letter “I”, alluding to the word “interview,” followed by a cardinal number, starting with the number “1”, referring to the order in which the interview was conducted.

### Study design

This is a descriptive and exploratory study, with a qualitative approach, in which the COnsolidated criteria for REporting Qualitative research instrument was used as a guide in the methodological process^(25)^.

### Study setting

The chosen starting point was a public university in the state of Rio de Janeiro, which offers a graduate course in stoma therapy nursing.

### Data source

The study population included entrepreneurial stoma therapists, recruited using the snowball method, in which the first research participants refer new participants, who in turn refer others, successively, until the proposed objective is reached or the point of saturation is reached, when the interviews no longer lead to an increase in new information^(26,27)^.

Eligible participants were stoma therapy nurses from any municipality in the country, regardless of gender, who were or had been involved in entrepreneurial activities in the specialty. This definition sought to encompass geographical and experiential diversity, ensuring representation of the multiple forms of entrepreneurial activity.

Entrepreneurial activities were considered to include actions in assistance, teaching, management, and research, including the creation of services, products, and technologies, consulting, training, and innovation in care processes. It is noteworthy that all participants held entrepreneurial positions at the time of data collection, allowing for a detailed exploration of the challenges, specificities, and potential of this professional profile in Brazil.

The initial selection of participants was intentional, motivated by the principal researcher’s close relationship with some stoma therapists who fit the study profile. Invitations were sent via messaging app or email, targeting faculty and graduates of the aforementioned course who were already recognized entrepreneurs in the field.

Initially, four stoma therapists were interviewed-three graduates and one faculty member-and, in each interview, they were asked to indicate at least one other professional with entrepreneurial experience. This snowball sampling strategy allowed the research to expand beyond the researcher’s initial circle. The data from the four initial interviews were fully included in the analysis, contributing to the construction of this study.

Based on the information provided by these initial participants, other interviewees were contacted, who, in turn, also indicated at least one entrepreneurial stoma therapist. Thus, the remaining participants were identified, totaling 26 entrepreneurial stoma therapists from four of the five regions of Brazil. It is noteworthy that no specialists from the North region were identified.

### Data collection and organization

Data collection, carried out by the author between January and April 2020, used semi-structured interviews with a two-part script. The first part was dedicated to characterizing participants, such as sex, age, and years of training. The second part included open-ended questions related to the object of the study, namely: i) discuss the entrepreneurial venture you developed; ii) discuss the difficulties that stoma therapy nurses encounter in exercising their entrepreneurial activity in this specialty; and iii) describe suggestions to leverage the entrepreneurial potential of stoma therapy nurses.

Four in-person interviews and 22 videoconference interviews were conducted, due to the geographical distance of some participants and the recommendations for social isolation during the COVID-19 pandemic declared by the World Health Organization on March 11, 2020.

The interviews lasted an average of 22 minutes and were recorded using a digital voice recording device, allowing full access to participants’ statements. All content was transcribed by the principal researcher without the use of software. The transcribed material was sent individually to participants for review and indication of any necessary corrections; however, no changes were deemed necessary, and the material proceeded to the analysis stage.

### Data analysis and treatment

The interviews were analyzed using content analysis techniques, which include the stages of pre-analysis, material exploration, and treatment and interpretation of results^(28)^. Initially, a preliminary reading of transcripts was conducted to immerse oneself in the content and identify relevant elements. Subsequently, the excerpts pertinent to the research objective were selected, and the content of the interviews was systematically organized, considering semantic and contextual aspects.

Manual data processing was chosen, without the use of software, allowing for a closer interaction between the principal researcher and the empirical material. Data were coded and organized into thematic categories based on the identification of recurring and relevant meanings, converting the raw content into results of interest for the study.

This process resulted in the collection of 351 Registration Units (RUs), which were grouped into two main categories: “Perceived difficulties in developing entrepreneurship in ostomy care” (233 RUs); and “Strategies to boost entrepreneurship in ostomy care” (118 RUs). It should be noted that there was no subdivision into subcategories, as the categories presented sufficient coherence and scope, providing clarity to the results.

## RESULTS

Of the 26 stoma therapists, 22 (84.62%) are female, and four (15.38%) are male. Concerning age, two (7.69%) participants were between 26 and 30 years old; ten (38.47%) were between 31 and 35 years old; four (15.38%) were between 36 and 40 years old; three (11.54%) were between 41 and 45 years old; one (3.85%) was between 46 and 50 years old; two (7.69%) were between 51 and 55 years old; and four (15.38%) were between 56 and 60 years old.

Regarding training as a stoma therapist, 11 (42.31%) had been practicing for one to five years, nine (34.61%) for six to ten years, two (7.69%) for 11 to 15 years, one (3.85%) for 16 to 20 years, and three (11.54%) for 26 to 30 years.

Most of experts interviewed (20) worked in an entrepreneurial way in capital cities (76.92%), while a smaller portion (06) worked in municipalities in the countryside (23.08%). In terms of regional distribution, a significant predominance was observed in the Southeast region, with 19 participants (73.08%), followed by the Central-West region, with three (11.54%), and the South (7.69%) and Northeast (7.69%) regions of Brazil, with two participants each.

The ventures ranged from initiatives more established in common sense, such as self-employment and home-based services, with the majority (14) of participants working in these areas, to product development, patents, and the creation of social institutions.

### Perceived difficulties in developing entrepreneurship in ostomy care

Several difficulties faced by stoma therapists in developing entrepreneurial projects were identified, such as the low appreciation and recognition of stoma therapists, the lack of knowledge about the specialty among the general population and other professionals, difficulties related to professional training, the entrepreneurial process, and financial issues.

In relation to the lack of appreciation and recognition of stoma therapy nurses by the population, the following statements characterized the analysis:

[...] *another obstacle is the social representation of nurses in society, where many do not identify nurses as autonomous professionals. Therefore, this remains a barrier for nurses to truly establish themselves as entrepreneurs.* (I15)

The incipient recognition of stoma therapists as an autonomous professional is also aggravated by the lack of knowledge on the part of the population and other healthcare professionals about the role, competencies, and skills of this specialist:

[...] *we had many obstacles with the medical community and other professions because they didn’t understand what stoma therapy was.* (I3)

[...] *I knew that my biggest challenge would be explaining what ostomy therapy is. It’s difficult to sell a service that nobody knows what it is or what it’s for.* (I4)

Another difficulty addressed was the lack of content related to entrepreneurship in undergraduate and graduate programs, with the training considered insufficient because the possibilities of entrepreneurship in this area were not comprehensively explored:

[...] *a nurse is not trained to be an entrepreneur. They are trained to be a nurse in the hospital, in the clinic* [...] *not to be entrepreneurial and innovative.* (I9)

[...] *I also see that, in our training, in our specialization, within the curriculum, I think there’s very little talk about entrepreneurship. I felt that the entrepreneurial aspect was sorely lacking. We only had one class, but it wasn’t enough.* (I23)

There are also evident difficulties related to the entrepreneurial process, including a lack of knowledge of the legal and administrative aspects involved in entrepreneurship:

[...] *it was simply a lack of knowledge. We didn’t even know how to legally open a business.* (I18)

[...] *you want to do it, but you don’t know where to start* [...] *and I think that’s the first difficulty for entrepreneurs* [...], *this bureaucratic part is very extensive.* (I23)

Participants also cited administrative issues related to health plans and insurance companies, for which there is no coverage related to payment for services provided by these professionals:

[...] *health insurance plans don’t value us. To be enrolled in a health insurance plan, you have to have a doctor behind.* [...]. *If you don’t have a doctor to provide that support, then you can’t register for the health plan.* (I11)

Another point mentioned concerns financial issues, whether due to a lack of resources or the difficulty professionals face in pricing and charging for their services.

[...] *I think money is the first thing that can inhibit a person from pursuing an entrepreneurial option* [...]. *So, I think the financial issue is the biggest obstacle.* (I10)

[...] *it’s really a financial issue, because the coverings are expensive, the materials are expensive, laser equipment is expensive* [...]. *Furthermore, we have difficulty charging for the service.* (I16)

Concerning financial issues, participants highlighted the charitable culture of the profession as a hindrance, both from patients’ and professionals’ own perspective.

[...] *people pay high prices for a doctor’s appointment, but they don’t want to pay for nursing care because they think we’ve always done it for free, and now we want to charge for what we do? They always see our service as charity.* (I4)

[...] *nurses have a cultural tendency to think they can’t charge for their services, and they don’t feel comfortable talking about money.* (I18)

### Strategies to boost entrepreneurship in ostomy care

As for the strategies listed to foster entrepreneurship in ostomy care, participants mentioned encouraging interaction among professionals themselves, through the exchange of experiences and support:

[...] *we need to spread this information* […]. *We should also create a network to obtain cheaper supplies. We should form an association of stoma therapy entrepreneurs.* (I23)

[...] *exchange ideas with other stoma therapists who have more expertise than you, so they can also start their own business and understand the path to avoid mistakes.* (I26)

Another strategy mentioned relates to the training, qualification, and scientific foundation of stoma therapists. Participants believe there needs to be more content on entrepreneurship offered in stoma therapy graduate courses.

[...] *I think we need to create more courses on this topic, and systematically introduce it into graduate studies.* (I3)

[...] *we need to include entrepreneurship as a subject in our training and courses for stoma therapy, because only then will we graduate with a minimum level of expertise.* (I18)

Participants also highlighted the need to broaden entrepreneurial vision, citing the incontinence sector as a promising area for starting a business. Institutional entrepreneurship, with intrapreneurial initiatives, was also emphasized.

[...] *I think we also don’t have to focus all our efforts on being business entrepreneurs* […], *It is necessary to create stoma therapy services within institutions so that nurses are recognized.* (I15)

[...] *when wound care professionals start their own businesses, they often think about opening clinics or businesses focused on wounds, but they forget about the increasingly prevalent issue of incontinence.* (I23)

## DISCUSSION

The lack of public knowledge about entrepreneurship in nursing and the undervaluation of the profession are obstacles pointed out in studies, in addition to the need for nurses themselves to understand the essence of entrepreneurial actions^(29,30)^. Furthermore, nurses themselves need to examine and grasp the essence of entrepreneurial actions within the profession^(10)^.

Stoma therapy, in turn, is a recent specialty in the country’s healthcare context, and there is still only superficial knowledge about the specialty and its areas of activity among patients and other healthcare professionals^(31)^.

In this sense, stoma therapists play a crucial role in valuing the specialty, promoting its dissemination and the sharing of knowledge to train more specialists, and broaden the understanding of the area by society and other healthcare professionals^(32)^. Thus, people will know who to look for or who to recommend in cases of needs related to the areas of practice of stoma therapy.

Difficulties related to entrepreneurial training were highlighted, and, regarding this issue, the literature emphasizes the need for a more in-depth approach to the topic^(8)^. There is a lack of incentives to awaken in nurses, from undergraduate studies onwards, the various modalities and fields of activity that this professional can explore, making them not only a good caregiver but also a successful entrepreneur^(33)^.

Nursing education still does not fully encompass entrepreneurship, requiring professionals to seek external courses or learn through practical experience, despite the demand for creativity, proactivity, and a forward-looking vision in the job market^(34)^. It is observed that educational institutions are not recognized as promoters of entrepreneurship in nursing, and that faculty and students are not sensitized to entrepreneurship, with few faculty members having the vision to encourage students to develop entrepreneurial activities^(1,27)^.

The incipient approach to entrepreneurship in undergraduate and graduate education creates obstacles for nurses, discouraging them, especially due to a lack of knowledge of legislation and information on the subject, which are considered major impediments to entrepreneurship^(27)^.

A study conducted in the United States with 102 nurses corroborates this analysis, indicating that they lack technical knowledge related to entrepreneurship, such as billing, legal regulations, and other practical issues^(35)^.

Thus, these difficulties can be associated with the limited approach to the topic of business and entrepreneurship in nursing education. It is therefore necessary for students to have access to an educational culture that is also oriented towards entrepreneurship, in order to increase entrepreneurial intent among nursing students^(36)^.

In addition to the limited knowledge on the subject, the complexity of bureaucratic and administrative processes was pointed out by participants, which is echoed in the literature^(10)^. Even knowing the licensing stages of their businesses, entrepreneurs still face difficulties, such as registration with legal bodies, long deadlines, and costs associated with documentation^(2,5)^.

Health plans and insurance companies also represent barriers to the entrepreneurship of stoma therapists by hindering patient access and limiting payments, reimbursements, and billing, due to the failure to recognize nurses as independent professionals because of laws and regulations that restrict their practice^(2,5,33)^.

In Brazil, newer professions such as physiotherapy, occupational therapy, psychology, nutrition, and speech therapy have already advanced into autonomous spaces of clinical practice, while nursing still faces normative and cultural restrictions to entrepreneurship^(37)^.

This issue requires political organization among nurses in order to seek the deregulation of the liberal practice of the profession, ensuring their autonomous practice supported by the competent bodies^(33)^.

The Federal Council of Nursing (In Portuguese, *Conselho Federal de Enfermagem* - COFEN) and the Brazilian Nursing Association (In Portuguese, *Associação Brasileira de Enfermagem*) work to provide this support for entrepreneurship in nursing through resolutions, guidelines, and training. COFEN Resolution 568/2018 establishes rules for opening private practices and providing independent services, defining legal limits, responsibilities, and ethical standards^(38)^.

Financial issues, such as high costs for taxes, equipment, supplies and infrastructure, rent or purchase of commercial premises, also constitute one of the barriers to entrepreneurship, according to experts, due to the fact that, for a business to grow and strengthen, adequate financial infrastructure is necessary^(27,10)^.

There is yet another complicating factor of a cultural nature: the issue of pricing and charging for services versus the charitable culture of the profession. It is clear that the difficulty in this case is related to both the population and the stoma therapist themselves. On one hand, there is society, which cannot understand nurses as someone who can charge for their services, and on the other hand, the professionals themselves, who do not feel comfortable charging the appropriate fee for their services, due to the charitable culture of nursing^(5,33)^.

Historically, nursing is perceived as a caring and charitable practice, making it difficult to understand it as an entrepreneurial activity in the business field, which can lead to a decrease in the valuation of the product of their work^(1,30)^. Many nurses, in fact, understand their work as being driven more by love than by financial considerations, as they are bound by cultural values deeply rooted in the profession^(5)^.

It is necessary for stoma therapists to overcome this altruistic nature of the profession and build an entrepreneurial mindset, in order to perceive themselves as an important professional in the job market and, in this way, show society the relevance of nurses’ work in the health sector^(1,30)^.

It is emphasized that it is possible to leverage entrepreneurship in this specialty through dissemination strategies, exchange of experiences, training of specialists, and broadening the vision of specialists’ entrepreneurial fields of action. From this perspective, participants’ statements reveal the importance of knowledge exchange between more experienced and less experienced entrepreneurs, in order to address doubts and increase the likelihood of success in the venture. This exchange of experience and knowledge can be done through technology, virtual networks, and mentoring, which enable the creation of new spaces for interaction and expand the possibilities of contact among entrepreneurs^(39)^.

It is important to maintain a network of relationships with more experienced entrepreneurs from the moment they begin planning their company to learn about the strategies adopted by the most experienced entrepreneurs, acquire the ability to make independent decisions, become more creative, innovative, and more attentive to opportunities, thus assessing the risks and adequately preparing for the challenges of their venture^(40)^.

There is also a need for stoma therapists to expand the topic of entrepreneurship in undergraduate and, especially, graduate courses in stoma therapy. Educational managers and faculty members need to pay special attention to this topic, incorporating entrepreneurial content into the curriculum, with a pedagogical methodology based on fostering creativity, initiative, and critical, reflective, and innovative thinking, thus expanding the business knowledge of these professionals^(1,41)^.

Entrepreneurial education is necessary to expand knowledge and skills in areas such as exploring ideas and opportunities, business planning, management techniques, marketing, finance, accounting, law, among others^(41,42)^.

In this sense, even professionals who do not intend to start their own business can be entrepreneurial in their workplaces, thus acting as intrapreneurs. Therefore, through intrapreneurship, the stoma therapist can offer differentiated and innovative services in healthcare settings, such as hospitals and clinics, assuming a variety of roles and gaining greater social and professional visibility^(2)^.

Therefore, stoma therapists who work in hospitals, clinics, and other healthcare services can also act entrepreneurially, and it is necessary to further promote this modality as a way to leverage their entrepreneurial potential.

Entrepreneurs, as already mentioned, are those who, among other things, are able to see opportunities. In this sense, even though stoma therapists who are entrepreneurs can already be considered visionaries within their own specialty, there are still areas in which they need to invest. The area of incontinence is a field within the specialty cited by the interviewees as deserving special attention for entrepreneurship, leading to reflection on the need for this field to be further explored by specialists^(17)^.

Considering the strategies mentioned, and in comparison with the international scenario, entrepreneurship in nursing, although new in some places, has been recognized as a strategic resource to face complex challenges and promote innovative advances in healthcare provision^(3,43)^. These experiences can offer practical ways to expand the autonomy and visibility of ostomy care and nursing as a whole in Brazil.

### Study limitations

This study is limited by the absence of stoma therapist participants from the Northern region of Brazil, which restricts the national representativeness of the findings and may limit the understanding of regional specificities related to entrepreneurship in stoma therapy. This geographical gap may influence the diversity of experiences reported, considering that socioeconomic, cultural, and structural factors vary significantly between regions of the country and impact entrepreneurial practice in nursing.

Another relevant methodological limitation concerns the use of the snowball sampling technique, in which initial participants indicate new subjects for the research. Although this strategy is useful for accessing specific and difficult-to-locate groups, it can generate biases arising from the homogeneity of the group formed, since contact networks tend to bring together individuals with similar trajectories, contexts, and perspectives. This bias potentially reduces the sample’s heterogeneity and, consequently, the breadth of perspectives captured.

It is therefore recommended that further research be conducted to minimize these gaps and to deepen discussions related to entrepreneurship in nursing, especially in the context of this specialty.

### Contributions to nursing, health, or public policy

It is believed that this work will serve as a subsidy for the performance of stoma therapy nurses who wish to undertake this practice, because when the main difficulties that may be encountered and the best strategies to overcome them are known in advance, it becomes possible to carry out better planning, closer to their reality, aiming at facilitating and improving the entire process.

With regard to nursing education, this study may serve as a basis for disseminating an entrepreneurial educational culture for specialization courses in stoma therapy, in addition to providing didactic support to students and stoma therapy professionals in developing their critical thinking about entrepreneurship, in nursing as a whole and in the specialty in question.

In relation to the contribution to nursing research, it can be inferred that this article may minimize the gap in scientific production on the subject, and may even encourage other studies that address entrepreneurship in other nursing specialties.

## FINAL CONSIDERATIONS

The results indicated that the difficulties and challenges of entrepreneurship in stoma therapy are undeniable, but the search for innovative solutions is imperative for the advancement and improvement of the specialty. The lack of appreciation and recognition of stoma therapists, the lack of knowledge about the specialty among the population and other professionals, the incipient knowledge about the legal and administrative process for entrepreneurship, financial issues, and the incipient nature of training on the subject are the main obstacles to be overcome.

Adopting the strategies mentioned in the study, such as the exchange of experiences and support among professionals, expanding the availability of content on the subject in nursing education and qualification, and broadening professionals’ vision regarding entrepreneurial possibilities in the specialty, can mitigate the challenges. Practical examples that can be adopted in daily professional practice include financial planning, strategic partnerships, the use of marketing tools, and the continuous pursuit of professional development.

To strengthen this trajectory, it is essential that higher education institutions integrate entrepreneurship into their curricula, promoting training more aligned with the contemporary demands of the profession. By proactively recognizing and addressing the barriers listed, stoma therapists and nursing professionals in general are not only shaping the future of their profession, but also contributing significantly to greater professional visibility and recognition.

## Data Availability

Not applicable.
