# Supplementary material for: Entrepreneurship in stoma care nursing: difficulties experienced and strategies for overcoming them
Source: Rev Bras Enferm. 2026 Aug 7;79:e20240242. doi: 10.1590/0034-7167-2024-0242 (PMC13450937; doi:10.1590/0034-7167-2024-0242)
Supplement: Translation

# Translation: Empreendedorismo na enfermagem em estomaterapia: dificuldades vivenciadas e estratégias de superação

**DOI:** 10.1590/0034-7167-2024-0242pt

**Authors:** Lívia Nunes Rodrigues Leme, Carolina Cabral Pereira da Costa, Eloá Carneiro Carvalho, Samira Silva Santos Soares, Bruno de Sousa Pappalardo, Gustavo Assis Afonso, Lana de Medeiros Escobar, Norma Valéria Dantas de Oliveira Souza

**Affiliations:** I Universidade do Estado do Rio de Janeiro. Rio de Janeiro, Rio de Janeiro, Brasil; II Universidade Estadual de Santa Cruz. Ilhéus, Bahia, Brasil

**Keywords:** Enfermagem, Estomaterapia, Empreendedorismo, Autonomia Profissional, Mercado de Trabalho.

## Abstract

**Objetivos:**

analisar as dificuldades vivenciadas por enfermeiros estomaterapeutas na realização de atividades empreendedoras na especialidade e discutir as estratégias de superação.

**Métodos:**

trata-se de estudo qualitativo, descritivo-exploratório, realizado com 26 estomaterapeutas empreendedores, captados através da técnica *snowball.* O tratamento dessas informações deu-se pela técnica de análise de conteúdo.

**Resultados:**

as dificuldades foram pouca valorização e reconhecimento profissional, desconhecimento da especialidade por outras categorias da saúde, e pouca familiaridade com os processos legais e administrativos para empreender. Questões financeiras e a lacuna de conteúdo na formação também se mostraram desafios significativos. Quanto às estratégias, elencaram-se troca de experiências entre os profissionais, ampliação da oferta de conteúdo sobre a temática e alargamento da visão do profissional sobre as possibilidades de empreendedorismo.

**Considerações Finais:**

é premente a implementação de estratégias para driblar as barreiras ao empreendedorismo em estomaterapia. Tal medida pode ampliar a possibilidade de sucesso profissional, visibilidade e valorização da enfermagem.

## INTRODUÇÃO

Historicamente, a enfermagem enfrenta desafios de valorização social, reflexo de um modelo hospitalocêntrico e centrado no médico^(1,2)^. Todavia, desde 2003, observa-se crescimento anual médio de 25% no número de estabelecimentos geridos por enfermeiros, sobretudo consultórios, clínicas especializadas e serviços domiciliares nas regiões Sul e Sudeste^(3)^.

Esse avanço da profissão indica uma transição em direção à interdisciplinaridade e reconfiguração dos papeis profissionais, reforçando a necessidade de práticas mais horizontais e colaborativas em benefício de indivíduos, famílias e comunidades^(4)^. Apesar disso, enfermeiros criativos e empreendedores ainda enfrentam resistência cultural, sendo frequentemente percebidos apenas como procedimentais, o que limita seu reconhecimento social e profissional^(5)^.

Destaca-se que a atuação de forma inovadora e empreendedora na enfermagem pode ser uma oportunidade de reconfigurar a profissão devido ao potencial de aumentar a autonomia profissional e a visibilidade da enfermagem. Ademais, caracteriza-se como uma forma de atender, de maneira diferenciada, pertinente e oportuna, às necessidades de saúde da população^(6-8)^.

A autonomia profissional se refere à capacidade do enfermeiro de tomar decisões e conduzir sua prática com base em conhecimentos técnicos, científicos, éticos e legais, abrangendo cuidado, gestão e educação em saúde. Enfermeiros empreendedores aplicam seu conhecimento clínico e gerencial desenvolvendo produtos, tecnologias e estratégias para melhoria de processos e do cuidado^(9)^.

Globalmente, o empreendedorismo na enfermagem cresce ao unir inovação, autonomia e impacto social. Profissionais têm explorado nichos como clínicas especializadas, atenção domiciliar, telenfermagem, *startups*, consultorias e soluções digitais^(10,11)^. Tais iniciativas, baseadas em evidências e ética, fortalecem a sustentabilidade dos sistemas de saúde, reposicionando a enfermagem como protagonista e promovendo melhorias na assistência e na experiência do paciente^(11)^.

O empreendedorismo pode ser entendido como um processo no qual um indivíduo vislumbra uma oportunidade e age sobre ela para criar valor, desenvolvendo algo novo ou aperfeiçoando algo já existente^(8,12)^. A literatura classifica o empreendedorismo por meio de três tipos: o empresarial ou de negócios, que se refere à criação de uma empresa ou um negócio para prática profissional autônoma^(13,14)^; o intraempreendedorismo, no qual empreendimento é desenvolvido em organizações públicas ou privadas nas quais o empreendedor já atua^(7,8,14)^; e o social, cujo foco está no uso de estratégias baseadas no mercado para gerar benefício social^(7,8,14)^.

Considerando essas definições e as características do perfil empreendedor, observa-se que o enfermeiro, seja generalista ou especialista, pode atuar de forma empreendedora nas três modalidades. Nesse contexto, a estomaterapia se destaca como uma especialidade promissora voltada ao cuidado de pessoas com estomias, feridas, incontinências anal e urinária, fístulas, tubos, e drenos^(15,16)^, abrindo amplas possibilidades para práticas inovadoras.

A estomaterapia tem crescido no Brasil, com predominância do cuidado assistencial, sobretudo no tratamento de feridas, seguido por estomias e incontinências, e maior concentração de profissionais nas regiões Sudeste, Nordeste e Sul, onde há mais cursos de formação^(17)^. Esse cenário impulsiona o empreendedorismo em áreas como consultórios, clínicas, *homecare*, consultorias, capacitação e desenvolvimento de tecnologias assistenciais^(17-19)^.

No Brasil, pesquisa realizada em 2016 estimou o número de 1.371 especialistas formados em cursos credenciados pela Associação Brasileira de Estomaterapia: Estomias, Feridas e Incontinências^(17)^. No cenário internacional, a estomaterapia também vem se consolidando como uma especialidade estratégica no cuidado em saúde, embora ainda haja escassez de dados sobre a força de trabalho em diferentes países. No Reino Unido, estima-se a atuação de cerca de 600 enfermeiros especialistas para atender mais de 176 mil pessoas com estomias, e na Espanha, quase metade dos hospitais públicos conta com estomaterapeutas^(20,21)^.

O estomaterapeuta dispõe de um amplo leque de oportunidades para empreender, abrangendo áreas como assistência, ensino, pesquisa, gestão, assessoria e consultoria técnica, tanto em serviços públicos quanto em privados, incluindo atendimento domiciliar, ambulatórios, clínicas e empresas de produtos médico-hospitalares^(15,16)^. No entanto, embora o campo seja promissor, ainda existem barreiras significativas, como limitações estruturais, burocráticas e culturais, que podem dificultar ou desestimular a adoção de práticas empreendedoras por esses profissionais^(5,12)^.

Nessa perspectiva, é necessário estudar as situações que dificultam a prática do empreendedorismo na enfermagem, bem como aprofundar as estratégias para superação dos obstáculos para o desenvolvimento de ações empreendedoras na profissão, sobretudo na especialidade em questão.

Destaca-se que as discussões sobre empreendedorismo na enfermagem ainda são subexploradas, evidenciando lacunas na abordagem dessa temática e, mais ainda, na estomaterapia^(8,22,23)^. Assim, o estudo tem relevância por estimular reflexões e debates, fortalecendo a cultura empreendedora e inovadora na enfermagem e na estomaterapia.

Este artigo é produto da dissertação de uma das autoras intitulada “Empreendedorismo na enfermagem em estomaterapia: potencializando oportunidades de trabalho”^(24)^.

## OBJETIVOS

Analisar as dificuldades vivenciadas por enfermeiros estomaterapeutas para realização de atividades empreendedoras na especialidade e discutir as estratégias de superação.

## MÉTODOS

### Aspectos éticos

A fim de atender o que determina a Resolução nº 466, de 12 de dezembro de 2012, do Conselho Nacional de Saúde, o presente estudo foi aprovado pelo Comitê de Ética em Pesquisa. Antes de cada entrevista, os participantes leram, assinaram e receberam uma cópia de um Termo de Consentimento Livre e Esclarecido. A fim de resguardar o sigilo da identidade dos entrevistados, criou-se uma codificação com a letra “E”, em alusão à palavra “entrevista”, seguida de um número cardinal, iniciando pelo número “1”, referente à ordenação da realização da entrevista.

### Tipo de estudo

Trata-se de estudo descritivo e exploratório, de abordagem qualitativa, no qual utilizou-se o instrumento *COnsolidated criteria for REporting Qualitative research* como guia no percurso metodológico^(25)^.

### Cenário do estudo

O cenário selecionado como ponto de partida foi uma universidade pública do estado do Rio de Janeiro, que oferece um curso de pós-graduação em enfermagem em estomaterapia.

### Fonte dos dados

A população do estudo incluiu estomaterapeutas empreendedores, captados a partir do método *snowball*, no qual os primeiros participantes da pesquisa indicam novos participantes, que apontam outros, sucessivamente, até que seja alcançado o objetivo proposto ou o ponto de saturação, quando as entrevistas não conduzem mais a um aumento de novas informações^(26,27)^.

Foram elegíveis enfermeiros estomaterapeutas de qualquer município do país, independentemente do gênero, que estivessem ou já tivessem estado envolvidos em atividades empreendedoras na especialidade. Essa definição buscou contemplar a diversidade geográfica e de experiências, garantindo representatividade das múltiplas formas de atuação empreendedora.

Consideraram-se atividades empreendedoras ações em assistência, ensino, gestão e pesquisa, incluindo criação de serviços, produtos e tecnologias, consultoria, capacitação, e inovação nos processos de cuidado. Ressalta-se que todos os participantes exerciam funções empreendedoras no momento da coleta, permitindo explorar detalhadamente desafios, especificidades e potencialidades desse perfil profissional no Brasil.

A seleção inicial dos participantes ocorreu de forma intencional, motivada pela proximidade da pesquisadora principal com alguns estomaterapeutas que se enquadravam no perfil do estudo. Os convites foram realizados por meio de aplicativo de mensagens ou correio eletrônico direcionados a docentes e egressos do referido curso que já eram reconhecidamente empreendedores na área.

Inicialmente, foram entrevistadas quatro estomaterapeutas - três egressas e uma docente - e, a cada entrevista, solicitou-se a indicação de pelo menos um outro profissional com atuação empreendedora. Essa estratégia de amostragem em cadeia (*snowball*) permitiu expandir o alcance da pesquisa para além do círculo inicial da pesquisadora. Os dados provenientes das quatro entrevistas iniciais foram integralmente incluídos na análise, contribuindo para construção deste estudo.

A partir das indicações dessas primeiras participantes, chegou-se a outros entrevistados que, por sua vez, também indicaram pelo menos um estomaterapeuta empreendedor. Assim, captaram-se os demais participantes, perfazendo um total de 26 estomaterapeutas empreendedores de quatro das cinco regiões do Brasil. Destaca-se que não foram indicados especialistas da região Norte.

### Coleta e organização dos dados

A coleta de dados, realizada pela autora entre janeiro e abril de 2020, utilizou entrevistas semiestruturadas com roteiro em duas partes, sendo a primeira destinada à caracterização dos participantes, como sexo, idade e tempo de formação. A segunda parte incluiu questões abertas referentes ao objeto do estudo, a saber: i) discorra sobre o empreendimento que você desenvolveu; ii) disserte sobre as dificuldades que os enfermeiros estomaterapeutas encontram para exercer sua atividade empreendedora na especialidade; e iii) descreva sugestões para alavancar o potencial empreendedor do enfermeiro estomaterapeuta.

Foram realizadas quatro entrevistas presenciais e 22 por videoconferência, devido à distância geográfica de alguns participantes e às recomendações de isolamento social durante a pandemia de COVID-19 decretada pela Organização Mundial da Saúde em 11 de março de 2020.

As entrevistas realizadas tiveram duração média de 22 minutos, tendo sido gravadas por meio de um dispositivo digital de gravação de voz, o que permitiu o acesso integral às falas dos participantes. Todo o conteúdo foi transcrito pela pesquisadora principal, sem a utilização de *softwares* para tal. O material transcrito foi encaminhado individualmente aos participantes, para conferência e indicação de eventuais correções, mas não foi indicada necessidade de alterações, de modo que o material seguiu para a etapa de análise.

### Análise e tratamento dos dados

O tratamento das entrevistas foi conduzido pela técnica de análise de conteúdo, que compreende as etapas de pré-análise, exploração do material, e tratamento e interpretação dos resultados^(28)^. Inicialmente, procedeu-se à leitura flutuante das transcrições, para imersão no conteúdo e identificação de elementos relevantes. Em seguida, realizaram-se a seleção dos trechos pertinentes ao objetivo da pesquisa e a organização sistemática do conteúdo das entrevistas, contemplando aspectos semânticos e de contexto.

Optou-se pelo processamento manual dos dados, sem a utilização de *softwares*, permitindo maior aproximação da pesquisadora principal com o material empírico. Os dados foram codificados e organizados em categorias temáticas a partir da identificação de significados recorrentes e relevantes, convertendo o conteúdo bruto em resultados de interesse para o estudo.

Esse processo resultou na captação de 351 Unidades de Registro (URs), as quais foram agrupadas em duas categorias principais: “Dificuldades percebidas para o desenvolvimento do empreendedorismo na estomaterapia” (233 URs); e “Estratégias para potencializar o empreendedorismo na estomaterapia” (118 URs). Ressalta-se que não houve subdivisão em subcategorias, pois as categorias apresentavam coerência e abrangência suficientes, conferindo clareza aos resultados.

## RESULTADOS

Dos 26 estomaterapeutas, 22 (84,62%) são do sexo feminino, e quatro (15,38%), do sexo masculino. Quanto à idade, dois (7,69%) participantes tinham entre 26 e 30 anos, dez (38,47%), entre 31 e 35 anos, quatro (15,38%), entre 36 e 40 anos, três (11,54%), entre 41 e 45 anos, um (3,85%), entre 46 e 50 anos, dois (7,69%), entre 51 e 55 anos, e quatro (15,38%), entre 56 e 60 anos.

Sobre a formação como estomaterapeuta, 11 (42,31%) tinham de um a cinco anos de formados, nove (34,61%), de seis a dez anos, dois (7,69%), de 11 a 15 anos, um (3,85%), de 16 a 20 anos, e três (11,54%), de 26 a 30 anos.

A maior parte dos especialistas entrevistados (20) atuava de forma empreendedora nas capitais (76,92%), enquanto uma parcela menor (06) atuava em municípios do interior (23,08%). Em termos de distribuição regional, observou-se predominância significativa na região Sudeste, sendo 19 participantes (73,08%), seguida pelas regiões Centro-Oeste, com três (11,54%), Sul (7,69%) e Nordeste (7,69%) do Brasil, com dois participantes cada.

Os empreendimentos variaram desde as iniciativas mais consolidadas no senso comum, como os negócios próprios e os atendimentos domiciliares, com a maioria (14) de participantes atuando nessas áreas, até desenvolvimento de produtos, patentes e criação de institutos sociais.

### Dificuldades percebidas para o desenvolvimento do empreendedorismo na estomaterapia

Apreenderam-se algumas dificuldades de atuação do estomaterapeuta para desenvolver projetos empreendedores, como a pouca valorização e o pouco reconhecimento do estomaterapeuta, o desconhecimento da especialidade por parte da população e de outros profissionais, e as dificuldades relacionadas à formação profissional, ao processo empreendedor e às questões financeiras.

Quanto à falta de valorização e reconhecimento do enfermeiro estomaterapeuta por parte da população, as seguintes falas caracterizaram a análise:

[...] *outro dificultador é a representação social do enfermeiro na sociedade, onde muitos não identificam o enfermeiro como um profissional autônomo. Então, isso ainda é um obstáculo para que o enfermeiro possa, de fato, se estabelecer como empreendedor.* (E15)

O incipiente reconhecimento do estomaterapeuta como um profissional autônomo também é agravado pelo desconhecimento por parte da população e de outros profissionais de saúde sobre a atuação, competências e habilidades desse especialista:

[...] *a gente teve muitos entraves com o corpo médico e com outras profissões, porque não entendiam o que era a estomaterapia.* (E3)

[...] *eu sabia que a minha grande dificuldade seria mostrar o que é a estomaterapia. É difícil vender um serviço que ninguém sabe o que é e para que serve.* (E4)

Outra dificuldade abordada foi o déficit de conteúdo relacionado ao empreendedorismo na graduação e na própria especialização, sendo a formação considerada insuficiente por não serem amplamente abordadas as possibilidades de empreendedorismo para a área:

[...] *o enfermeiro não é preparado para ser um empreendedor. Ele é preparado para ser um enfermeiro do hospital, do posto* [...] *não para ser empreendedor e inovador.* (E9)

[...] *eu vejo, inclusive, que, na nossa formação, na nossa especialização, dentro da grade curricular, acho que fala muito pouco sobre empreendedorismo. Eu achei que a parte empreendedora faltou muito. A gente teve apenas uma aula, mas insuficiente.* (E23)

Evidenciam-se ainda dificuldades relacionadas ao processo empreendedor, sendo citado o desconhecimento dos aspectos legais e administrativos que envolvem o empreendedorismo:

[...] *a falta de conhecimento mesmo. A gente não sabia nem como abrir legalmente um negócio.* (E18)

[...] *você quer fazer, mas você não sabe por onde começar* [...] *e eu acho que essa é a primeira dificuldade do empreendedor* [...], *essa parte burocrática é muito grande.* (E23)

Os participantes também citaram questões administrativas relacionadas aos planos e às seguradoras de saúde, para os quais não há uma cobertura relacionada ao pagamento dos serviços prestados por esses profissionais:

[...] *os planos de saúde não nos valorizam. Para você ser contratado de um plano de saúde, você tem que ter um médico por trás* [...]. *Se você não tem um médico para dar esse respaldo, então você não consegue se cadastrar no plano de saúde.* (E11)

Outro ponto citado diz respeito a questões financeiras, seja pela escassez de recursos, seja pela escassez de recursos ou pela dificuldade que o próprio profissional enfrenta para precificar e cobrar por seus serviços.

[...] *acho que o dinheiro é o primeiro que pode inibir a pessoa de buscar uma opção empreendedora* [...]. *Então, acho que a questão financeira é o maior obstáculo.* (E10)

[...] *a questão financeira mesmo, porque as coberturas são caras, os materiais são caros, aparelho de laser é caro* [...]. *Além disso, temos dificuldade para cobrar o serviço.* (E16)

Ainda sobre as questões financeiras, os participantes destacaram a cultura caritativa da profissão como um dificultador, tanto pela visão do paciente quanto do próprio profissional.

[...] *as pessoas pagam altos valores numa consulta médica, mas não querem pagar para enfermagem, porque eles pensam que a gente sempre fez isso de graça, e agora vamos querer cobrar pelo que fazemos? Eles sempre encaram nosso serviço como caridade.* (E4)

[...] *o enfermeiro tem uma coisa cultural de achar que não pode cobrar pelo que faz, de não se sentir à vontade para falar sobre dinheiro.* (E18)

### Estratégias para potencializar o empreendedorismo na estomaterapia

Com relação às estratégias elencadas para fomentar o empreendedorismo na estomaterapia, os participantes citaram o incentivo entre os próprios profissionais, com troca de experiências e apoio:

[...] *a gente precisa disseminar essas informações* […]. *Também a gente deveria montar uma rede para obter insumos mais baratos. Montar uma associação de empreendedores estomaterapeutas.* (E23)

[...] *trocar com outros estomaterapeutas que tenham mais expertise que você para também empreender, entender como é a trajetória para evitar equívocos.* (E26)

Outra estratégia citada se relaciona à formação, à capacitação e ao embasamento científico dos estomaterapeutas. Os participantes acreditam que é necessário haver maior oferta de conteúdo sobre o empreendedorismo nos cursos de pós-graduação de estomaterapia:

[...] *eu acho que a gente tem que fazer mais cursos com essa temática, introduzir sistematicamente essa temática na pós-graduação.* (E3)

[...] *a gente precisa ter na formação e no curso de estomaterapia a disciplina de empreendedorismo, porque só assim a gente vai sair com uma bagagem mínima.* (E18)

Os participantes também destacaram a necessidade de ampliar a visão empreendedora, citando a área das incontinências como um setor promissor para iniciar um empreendimento. Destacou-se também o empreendimento institucional, com ações intraempreendedoras.

[...] *eu acho que a gente também não tem que focar todos os nossos esforços em ser empreendedor de negócios* […], *é preciso criar serviços de estomaterapia dentro das instituições para que o enfermeiro seja reconhecido.* (E15)

[...] *os estomaterapeutas, quando vão empreender, já pensam logo em abrir clínicas, abrir seu negócio voltado para feridas, mas esquecem essa parte da incontinência, que tem crescido cada vez mais.* (E23)

## DISCUSSÃO

A falta de conhecimento da população sobre o empreendedorismo em enfermagem e a subvalorização da profissão são obstáculos apontados em estudos, além da necessidade de o próprio enfermeiro compreender a essência das ações empreendedoras^(29,30)^. Além disso, o próprio enfermeiro tem necessidade de examinar e apreender a essência das ações empreendedoras na profissão^(10)^.

A estomaterapia, por sua vez, é uma especialidade recente no contexto de saúde do país, havendo ainda um conhecimento superficial sobre a especialidade e seus campos de atuação em relação aos pacientes e aos outros profissionais de saúde^(31)^.

Nesse sentido, o estomaterapeuta desempenha papel crucial na valorização da especialidade, promovendo sua divulgação e socialização do conhecimento para formar mais especialistas, e ampliar a compreensão da área pela sociedade e por outros profissionais de saúde^(32)^. Assim, as pessoas saberão a quem procurar ou quem indicar em casos de necessidades relacionadas às áreas de atuação da estomaterapia.

Dificuldades relacionadas à formação empreendedora foram salientadas, e, em relação a essa questão, a literatura destaca que é mister uma abordagem mais aprofundada sobre o tema^(8)^. Existe uma carência de incentivos para despertar nos enfermeiros, desde a graduação, as diversas modalidades e os vários campos de atuação que esse profissional pode explorar, tornando-o não apenas um bom assistencialista, mas também um empreendedor de sucesso^(33)^.

A formação em enfermagem ainda não contempla plenamente o empreendedorismo, exigindo que os profissionais busquem cursos externos ou aprendam na prática, apesar da demanda por criatividade, proatividade e visão de futuro no mercado de trabalho^(34)^. Constata-se que as instituições formadoras não são reconhecidas como incentivadoras do empreendedorismo na enfermagem e que docentes e discentes não estão sensibilizados para o empreendedorismo, sendo poucos os docentes com visão para incentivar o discente a desenvolver atividades empreendedoras^(1,27)^.

A abordagem incipiente do empreendedorismo na graduação e pós-graduação cria obstáculos aos enfermeiros, desestimulando-os, especialmente devido ao desconhecimento da legislação e à falta de informações sobre o tema, que são considerados grandes dificultadores do empreendedorismo^(27)^.

Estudo realizado nos Estados Unidos da América com 102 enfermeiros corrobora essa análise, apontando que esses carecem de conhecimentos técnicos relacionados ao empreendedorismo, como faturamento, normas legais e outras questões práticas^(35)^.

Assim, essas dificuldades podem ser associadas à pouca abordagem do tema de negócios e empreendedorismo na formação do enfermeiro. Faz-se necessário, portanto, o acesso dos estudantes a uma cultura educacional orientada também para o empreendedorismo, de forma a ampliar a intenção empreendedora entre os estudantes de enfermagem^(36)^.

Além do conhecimento incipiente sobre o tema, a complexidade dos processos burocráticos e administrativos foi apontada pelos participantes, o que encontra eco na literatura^(10)^. Mesmo conhecendo as etapas de licenciamento dos seus negócios, os empreendedores ainda enfrentam dificuldades, como registros em órgãos legais, longos prazos e custos com documentação^(2,5)^.

Planos e seguradoras de saúde também representam barreiras ao empreendedorismo do estomaterapeuta ao dificultar o acesso dos pacientes e limitar pagamentos, reembolsos e cobranças, por não se reconhecer o enfermeiro como um profissional autônomo devido às leis e resoluções que restringem sua atuação^(2,5,33)^.

No Brasil, profissões mais jovens, como fisioterapia, terapia ocupacional, psicologia, nutrição e fonoaudiologia, já avançaram em espaços autônomos de prática clínica, enquanto a enfermagem ainda enfrenta as restrições normativas e culturais ao empreendedorismo^(37)^.

Essa questão necessita de organização política dos enfermeiros, no sentido de buscar a desburocratização do exercício liberal da profissão, assegurando sua atuação autônoma respaldada pelos órgãos competentes^(33)^.

O Conselho Federal de Enfermagem (COFEN) e a Associação Brasileira de Enfermagem atuam de forma a oferecer esse suporte ao empreendedorismo na enfermagem por meio de resoluções, orientações e capacitações. A Resolução COFEN nº 568/2018 estabelece normas para abertura de consultórios e prestação de serviços autônomos, definindo limites legais, responsabilidades e padrões éticos^(38)^.

As questões financeiras, como altos custos com tributos, equipamentos, insumos e infraestrutura, aluguéis ou compra de salas comerciais, também constituem uma das barreiras ao empreendedorismo, segundo os especialistas, devido ao fato de que, para o empreendimento crescer e se fortalecer, é necessária uma infraestrutura financeira adequada^(27,10)^.

Há ainda mais um dificultador de cunho cultural: a questão da precificação e cobrança de serviços *versus* a cultura caritativa da profissão. Percebe-se que a dificuldade nesse caso está relacionada tanto à população quanto ao próprio estomaterapeuta. De um lado, está a sociedade, que não consegue compreender o enfermeiro como alguém que pode cobrar por seus serviços, e de outro, o próprio profissional, que não se sente à vontade em cobrar o valor devido por seus serviços, devido à cultura caritativa da enfermagem^(5,33)^.

Historicamente, a enfermagem é percebida como uma prática assistencialista e caritativa, sendo difícil entendê-la como uma atividade empreendedora na área dos negócios, o que pode ocasionar uma diminuição da valorização do produto do seu trabalho^(1,30)^. Muitos enfermeiros, inclusive, possuem uma compreensão de trabalhar mais por amor do que por questões financeiras, pois estão presos a valores culturais arraigados na profissão^(5)^.

É necessário que o estomaterapeuta supere essa natureza altruísta da profissão e construa uma mentalidade empreendedora, no sentido de conseguir se perceber como um profissional importante no mercado de trabalho e, dessa forma, mostrar à sociedade a relevância do trabalho realizado pelos enfermeiros no setor da saúde^(1,30)^.

Salienta-se que é possível alavancar o empreendedorismo na especialidade por meio de estratégias de divulgação, troca de experiências, capacitação dos especialistas e ampliação da visão dos campos de ação empreendedora do especialista. Nessa perspectiva, verifica-se, nas falas dos participantes, a importância da troca de conhecimento entre os empreendedores mais experientes e os menos experientes, a fim de sanar dúvidas e ampliar a possibilidade de sucesso no empreendimento. Tal troca de experiência e conhecimento pode ser feita através das tecnologias, das redes virtuais e de mentorias, que possibilitam a criação de novos espaços de interação e ampliam as possibilidades de contato entre os empreendedores^(39)^.

É importante manter uma rede de relações com empreendedores mais experientes desde o momento em que se inicia o planejamento da empresa, para conhecer as estratégias adotadas pelos mais experientes, adquirir a capacidade de tomar decisões independentes, tornar-se mais criativo, inovador e mais atento às oportunidades, avaliando, assim, os riscos e se preparando adequadamente para os desafios de seu empreendimento^(40)^.

Constata-se ainda a necessidade de os estomaterapeutas ampliarem, nos cursos de graduação e, principalmente, de pós-graduação em estomaterapia, a temática do empreendedorismo. Os gestores pedagógicos e docentes necessitam dar atenção especial à temática, trazendo para a formação o conteúdo do empreendedorismo, com metodologia pedagógica pautada no fomento à criatividade, iniciativa e capacidade crítica, reflexiva e inovadora, ampliando, assim, o conhecimento empresarial dos profissionais^(1,41)^.

Faz-se necessária uma educação empreendedora, para ampliar o conhecimento e as habilidades em áreas como exploração de ideias e oportunidades, planejamento de negócios, técnicas de gerenciamento, *marketing*, finanças, contabilidade, direito, entre outras^(41,42)^.

Nesse sentido, mesmo os profissionais que não têm intenções de empreender podem ser empreendedores nos seus locais de trabalho, agindo, portanto, como intraempreendedores. Assim, por meio do intraempreendedorismo, o estomaterapeuta pode oferecer serviços diferenciados e inovadores em ambientes de saúde, como os hospitais e clínicas, assumindo variedade de papéis e obtendo maior visibilidade social e profissional^(2)^.

Sendo assim, o estomaterapeuta que atua em hospitais, clínicas e demais serviços de saúde pode também atuar de forma empreendedora, sendo necessária maior difusão dessa modalidade também como uma forma de alavancar seu potencial empreendedor.

O empreendedor, como já foi citado, é aquele que, entre outras coisas, consegue enxergar as oportunidades. Nesse sentido, mesmo que os estomaterapeutas que empreendem já possam ser considerados visionários dentro da própria especialidade, ainda existem campos em que os mesmos precisam investir. A área da incontinência é um campo da especialidade citado pelos entrevistados como merecedor de especial atenção para o empreendedorismo, levando à reflexão sobre a necessidade de esse campo ser mais explorado pelos especialistas^(17)^.

Considerando as estratégias citadas, e em comparação com o cenário internacional, o empreendedorismo em enfermagem, apesar de novo em alguns locais, tem sido reconhecido como um recurso estratégico para enfrentar desafios complexos e promover avanços inovadores na prestação do cuidado em saúde^(3,43)^. Essas experiências podem oferecer caminhos práticos para ampliar a autonomia e visibilidade da estomaterapia e da enfermagem como um todo no Brasil.

### Limitações do estudo

Este estudo apresenta como limitação a ausência de participantes estomaterapeutas provenientes da região Norte do Brasil, o que restringe a representatividade nacional dos achados, podendo limitar a compreensão de especificidades regionais relacionadas ao empreendedorismo na estomaterapia. Tal lacuna geográfica pode influenciar a diversidade de experiências relatadas, considerando que fatores socioeconômicos, culturais e estruturais variam significativamente entre as regiões do país, impactando a prática empreendedora na enfermagem.

Outra limitação metodológica relevante se refere à utilização da técnica de amostragem *snowball* (amostragem em cadeia), na qual os participantes iniciais indicam novos sujeitos para a pesquisa. Embora essa estratégia seja útil para acessar grupos específicos e de difícil localização, ela pode gerar vieses decorrentes da homogeneidade do grupo formado, já que as redes de contato tendem a reunir indivíduos com trajetórias, contextos e perspectivas semelhantes. Esse viés potencialmente reduz a heterogeneidade da amostra e, consequentemente, a amplitude das visões captadas.

Recomenda-se, portanto, que novas pesquisas possam ser realizadas para minimizar essas lacunas e para aprofundar as discussões relacionadas ao empreendedorismo na enfermagem, especialmente no contexto da especialidade em tela.

### Contribuições para as áreas da enfermagem, saúde ou políticas públicas

Acredita-se que este trabalho servirá de subsídio para atuação do enfermeiro estomaterapeuta que deseja empreender, porque, quando se conhecem de antemão as principais dificuldades pelas quais se poderá passar e as melhores estratégias para superá-las, torna-se possível realizar um melhor planejamento mais próximo de sua realidade, visando à facilitação e melhoria de todo o processo.

Com relação ao ensino em enfermagem, este estudo poderá servir de base para disseminação de uma cultura educacional empreendedora para os cursos de especialização em estomaterapia, além de servir de apoio didático aos estudantes e profissionais estomaterapeutas no desenvolvimento de seu pensamento crítico acerca do empreendedorismo na enfermagem como um todo e na especialidade em tela.

Referente à contribuição para a pesquisa em enfermagem, infere-se que este artigo poderá minimizar a lacuna de produção científica sobre a temática, inclusive incentivando outros estudos que abordem o empreendedorismo em outras especialidades da enfermagem.

## CONSIDERAÇÕES FINAIS

Os resultados apontaram que as dificuldades e os desafios do empreendedorismo na estomaterapia são inegáveis, mas a busca por soluções inovadoras é imperativa para o avanço e aprimoramento da especialidade. A falta de valorização e reconhecimento do estomaterapeuta, o desconhecimento da especialidade pela população e por outros profissionais, o incipiente conhecimento sobre o processo legal e administrativo para empreender, as questões financeiras, e a incipiência na formação sobre o tema são os principais obstáculos a serem superados.

Adotar as estratégias mencionadas no estudo, como a troca de experiências e suporte entre os profissionais, ampliação da oferta de conteúdo sobre a temática na formação e qualificação do enfermeiro, e o alargamento da visão do profissional quanto às possibilidades de empreendedorismo na especialidade, podem mitigar os desafios. Exemplos práticos que podem ser adotados no cotidiano profissional se referem ao planejamento financeiro, parcerias estratégicas, uso de ferramentas de marketing, bem como busca contínua por atualização profissional.

Para fortalecer essa trajetória, é essencial que as instituições de ensino superior integrem o empreendedorismo em seus currículos, promovendo uma formação mais alinhada às demandas contemporâneas da profissão. Ao reconhecer e abordar proativamente as barreiras elencadas, os estomaterapeutas e profissionais de enfermagem, de uma forma geral, estão não apenas moldando o futuro da sua profissão, mas também contribuindo de maneira significativa para a maior visibilidade e valorização profissional.

## Data Availability

Não se aplica.
